# Development of a Novel Multiplex Immunoassay Multi-cruzi for the Serological Confirmation of Chagas Disease

**DOI:** 10.1371/journal.pntd.0004596

**Published:** 2016-04-01

**Authors:** Elodie Granjon, Marie-Laure Dichtel-Danjoy, Esber Saba, Ester Sabino, Lea Campos de Oliveira, Maan Zrein

**Affiliations:** 1 InfYnity Biomarkers, Lyon, France; 2 Faculdade de Medicina da USP, Dep de Molestias Infecciosas e Parasitárias, São Paulo, Brazil; US Food and Drug Administration, UNITED STATES

## Abstract

**Background:**

Chagas disease is due to the parasite *Trypanosoma cruzi*, a protist disseminated by a Triatome vector. This disease is endemic to Latin America and considered by WHO as one of the 17 world’s neglected diseases. In Europe and in North America, imported cases are also detected, due to migration of population outside of the endemic region. Diagnosis of *T*. *cruzi* infection is usually made indirectly by the detection of specific antibodies to *T*. *cruzi* antigens. Following initial diagnostic evaluation or screening test (qualifying or discarding blood donation), a confirmation test is performed for samples initially reactive. The test presented in this study aims at the confirmation/refutation of the infectious status of human blood samples and will permit taking appropriate clinical measures.

**Methodology/Principal Findings:**

We designed a novel array of twelve antigens and printed these antigens onto 96-well plates. We tested 248 positive samples *T*. *cruzi*, 94 unscreened blood donors’ samples from non-endemic area, 49 seronegative blood donors, 7 false-positive and 3 doubtful samples. The observed reactivities were analyzed to propose a decision-tree algorithm that correctly classifies all the samples, with the potential to discriminate false-positive results and sticky samples. We observed that antibodies levels (Sum of all antigens) was significantly higher for PCR positive than for PCR negative samples in all studied groups with Multi-cruzi.

**Conclusion/Significance:**

The results described in this study indicate that the Multi-cruzi improves the serological confirmation of Chagas disease. Moreover the “sum of all antigens” detected by Multi-cruzi could reflect parasitemia level in patients–like PCR signals does—and could serve as an indicator of parasite clearance in longitudinal follow-ups. Validation of this assay is still required on an independent large collection of well characterized samples including typical false-reactive samples such as Leishmaniasis.

## Author Summary

Eight million people are affected by Chagas disease worldwide, almost exclusively in South America. This chronic disease is initially asymptomatic but turns out to be life threatening in 30% of the cases, years after the infection. Two therapies are available, but their efficacy is maximal if the treatment is initiated shortly after contamination. In the absence of reliable symptoms, the *in vitro* diagnostic of Chagas Disease is thus a crucial tool to implement accurate clinical management. In order to permit universal access and a reliable confirmation of Chagas’s parasite presence, we have developed a new multiplex platform using a large number of selected antigens on easy handling 96 well ELISA microplates. In this manuscript, we present the first optimization data of the Multi-cruzi test that shows promising results for Chagas disease confirmation.

## Introduction

Chagas disease is life-threatening condition affecting 8 to 10 million persons in the world, predominantly in Latin America where the disease is endemic [1]. It is considered by WHO as one of the 17 world’s neglected diseases. In Europe and in North America, imported cases are also detected, due to migration of population outside of the endemic region [2]. The agent of the disease is the parasite *Trypanosoma cruzi*, a protist disseminated by a Triatome vector. Transmission also occurs by oral route, blood transfusion or congenitally. Chagas' disease is initially a silent disease that develops through different stages. Following *T*. *cruzi* exposure, patients enter the acute phase, during which the level of circulating parasites is high. Despite this exposure the symptoms are generally rare or benign (e.g., fever and occasionally an inflammatory reaction at the bite site). After the acute phase which lasts few months, most patients enter chronic Chagas disease. During this phase, parasites are less abundant and may be confined to certain host tissues like muscle or fat [3]. About 70% of chronic patients will never develop severe clinical complications. These patients have the indeterminate form of chronic Chagas disease [4]. However, approximately 30% of the patients, will suffer life threatening cardiac disorders, digestive, neurological or mixed alterations after a latency period ranging from 10 to 30 years.

As there is no vaccine, prevention of Chagas disease is made through control of the parasite vector’s spreading: house improvements, personal protection to prevent vector infestation or good hygiene practices in particular regarding the fresh fruits preparation. Moreover in order to prevent infection, screening of blood and organs donors, or new-born and children of infected mothers is essential to provide early diagnosis and treatment [5].

Different tests are available for the diagnosis of Chagas disease in the clinic or for screening of blood donations. Direct detection of parasite in the blood is performed by microscopy, haemoculture, xenodiagnosis or detection of the parasite’s nucleic acids. These assays are highly specific but lack sensitivity in the chronic stage during which parasites load in the blood is reduced [6]. Hence, diagnosis of *T*. *cruzi* infection is usually made indirectly by the detection of specific antibodies to *T*. *cruzi* antigens. FDA approved ELISA tests including either crude antigens (Ortho *T*. *cruzi* ELISA) or a mix of fusion proteins (Abbott PRISM Chagas [7]). To accurately determine the infectious status of a patient, two or three conventional tests based on different antigens are generally performed [8].

In a systematic review, Brasil et al. evaluated that commercial ELISAs have a pooled sensitivity of 99.3% (97.9%–99.9%) and a pooled specificity of 97.5% (88.5%–99.5%) [9]. The nonspecific reactions, causing inconclusive or false-positive results can explain the low specificity. Indeed, various pathogens from the trypanosomatid family (e.g., *Leishmania spp*. and *T*. *cruzi*) share a similar antigenic repertoire with common epitopes that can induce the production of cross-reactive antibodies [10]. Samples with an indeterminate result or low-level reactivity also present challenges for blood screening and when estimating prevalence or incidence rates for epidemiological surveillance [11]. In this context, the development of an immunoassay for detecting circulating antibodies against *T*. *cruzi* with a good performance appears to be crucial for efficient clinical management of Chagasic patients.

Following initial diagnostic evaluation or screening test (qualifying or discarding blood donation), a confirmation test is performed for samples initially reactive. This test will allow the confirmation/refutation of the infectious status of the human blood samples and will permit taking appropriate clinical measures.

Confirmation of infectious disease are classically performed through Western Blot or Dot Blot tests when available, allowing detection of discrete reactivities on specific antigens. FDA approved the usage of the Abbott ESA Chagas Dot Blot test that includes the same recombinant antigens as the Abbott ARCHITECT screening automate [12]. Some homemade tests such as TESA blots (Trypanosoma Excreted secreted antigens) are also used in some countries of Latin America for confirmation [13] [14]. The membrane-based multiparametric approach for the serology has proved efficient but incompatible with automation, therefore the INNO-LIA Chagas test, although validated, has never been made widely available [15] [16]. Taking into consideration the complexity of Chagas serological interpretation and the difficult implementation of Dot Blot technology in low income countries, we developed a multiplex ELISA-based protein array for the confirmation of Chagas disease. In this manuscript we present this innovative confirmation assay Multi-cruzi carried out with 12 different antigens printed on a 96-well plate. This assay is based on the discrete estimation of antibodies diversity allowing an accurate conclusion on the patient infectious status.

## Materials and Methods

### Ethics Statement

The experimental plan of the research study was in agreement with the Declaration of Helsinki and was approved by a local institutional ethical committee for each sampling site. Written informed consent were obtained for patients or blood donors after a detailed explanation on the usage for research purpose of their donation.

### Study Population and Samples Origin

In this study, samples originated from different center and countries (Table 1).

**Table 1:** Patient’s characteristic.

Sampling Site	Status	N	Gender F/M	Age, mean years (SD)	PCR Pos/Neg	Treatment Yes/No
BST, Barcelona	Infected Blood Donors	48	31/17	38 (10.74)	14/34	Unknown
	Control populations	49	Unknown	Unknown	N.D.	N.A.
	False-Positive*	7	4/3	44 (7.74)	0/7	N.A.
	Doubtful*	3	2/1	40 (11.05)	0/3	N.A.
USP, Brazil	SAMI Trop Cardiomyopathy Cohort	200	63/137	56 (11.82)	65/129	100/100
EFS, France	Controls populations	94	42/52	42 (15.63)	Untested	N.A.

* see Table 2 for detailed description of these samples

#### EFS (France)

We obtained 94 samples from the Etablissement Français du Sang (EFS) under cession agreement 20120224 after reviewing of the research protocol. The 94 blood donors tested in France were “not at risk” blood donors, meaning they are neither originating from, nor have travelled to, the endemic zone. Consequently their blood donations were not tested for Chagas disease, in conformity with local legislation.

#### BST (Barcelona, Spain)

107 samples were obtained from consenting blood donors enrolled at the Catalonian blood bank “Banc de Sang i Teixits”, (BST) of which 48 positive, 49 negative, 3 doubtful and 7 false-positive samples. All samples were initially screened on ARCHITECT ELISA for the Chagas disease. PCR was also performed on each sample in case of positivity on ARCHITECT ELISA (see PCR analysis section).

The 48 Infected Blood donors were all originating from the endemic zone (34 from Bolivia, 5 from Argentina, 2 from Paraguay, one from Brazil, Chile, Colombia, Equator, Uruguay and 2 of unknown origin). They were screened and confirmed positive on three different assays (Abbott, Ortho and Biokit).

The three Doubtful and seven false-positive samples with inconsistent results (see Table 2) on three different assays (Abbott, Ortho and Biokit ELISAs) were obtained from donors of the endemic zone (3 from Bolivia and 1 from Argentina) or from Spain (6 samples). Samples were considered as false-positive if reactive only on the first screening assay (Abbott) and negative on two consecutive assays (Ortho and Biokit ELISAs). Doubtful samples were reactive on two out of three tests (either Abbott and Biokit or Abbott and Ortho).

**Table 2:** False-positive (FP) and doubtful samples.

Groups	IB	Age	Gender	Origin	S/Co Architect	S/Co Architect Repeat	S/Co Elisa Biokit	S/Co Elisa Ortho	Multi-cruzi	*T*.*Cruzi* PCR
FP	3518	43	M	Spain	1.89	1.91	0.03	0.03	Neg	NEG
FP	3519	33	F	Spain	1.33	1.55	0.02	0.07	Neg	NEG
FP	3520	52	F	Spain	0.97	1.00	0.02	0.02	Neg	NEG
FP	3521	43	F	Bolivia	1.07	1.08	0.21	0.35	Neg	NEG
FP	3522	56	M	Spain	1.68	1.53	0.04	0.09	Neg	NEG
FP	3523	41	M	Spain	2.01	1.99	0.13	0.23	Neg	NEG
FP	3524	39	F	Spain	2.53	2.66	0.07	0.33	Neg	NEG
Doubtful	3515	27	F	Argentina	1.26	ND	4.77	0.13	Neg	NEG
Doubtful	3516	44	M	Bolivia	1.95	ND	0.72	0.96	Neg	NEG
Doubtful	3517	48	F	Bolivia	1.89	ND	2.53	0.70	Pos	NEG

Forty nine negative samples on Architect were also included in the analysis.

Investigational protocol PR(CS)373/2014 was approved by a local Ethical Comity (Hospital Val d’Hebron, Spain).

#### Sami trop Cohort (Sao Paulo, Brazil)

The Brazilian samples were collected as part of the NIAID funded Samitrop Project [17]. This cohort study is recruiting approximately 2,000 Chagas cardiomyopathy patients in 21 cities of the northern part of Minas Gerais state in Brazil. We randomly tested samples among patients with no previous treatment (n = 100) or with history of Benznidazole treatment ended more than ten years ago (n = 100). Study protocol was approved by Conep and by the University of Sao Paulo Medical IRB (ref 00580612.8.0000.0065).

### PCR Analysis

Regarding blood donor samples, qPCR analysis was performed as described by Piron et al [18]. Briefly, after standard DNA extraction, qPCR was performed using primers specific to Satellite sequences of *T*. *cruzi*. Human RNase P gene was used as an internal control of amplification.

PCR on the Cardiomyopathy cohort was performed on minicircle DNA using Tc-S36 and Tc-S35/A as probes [19].

### Novel Multiplex Chagas Assay: Multi-cruzi

**Fig 1:**
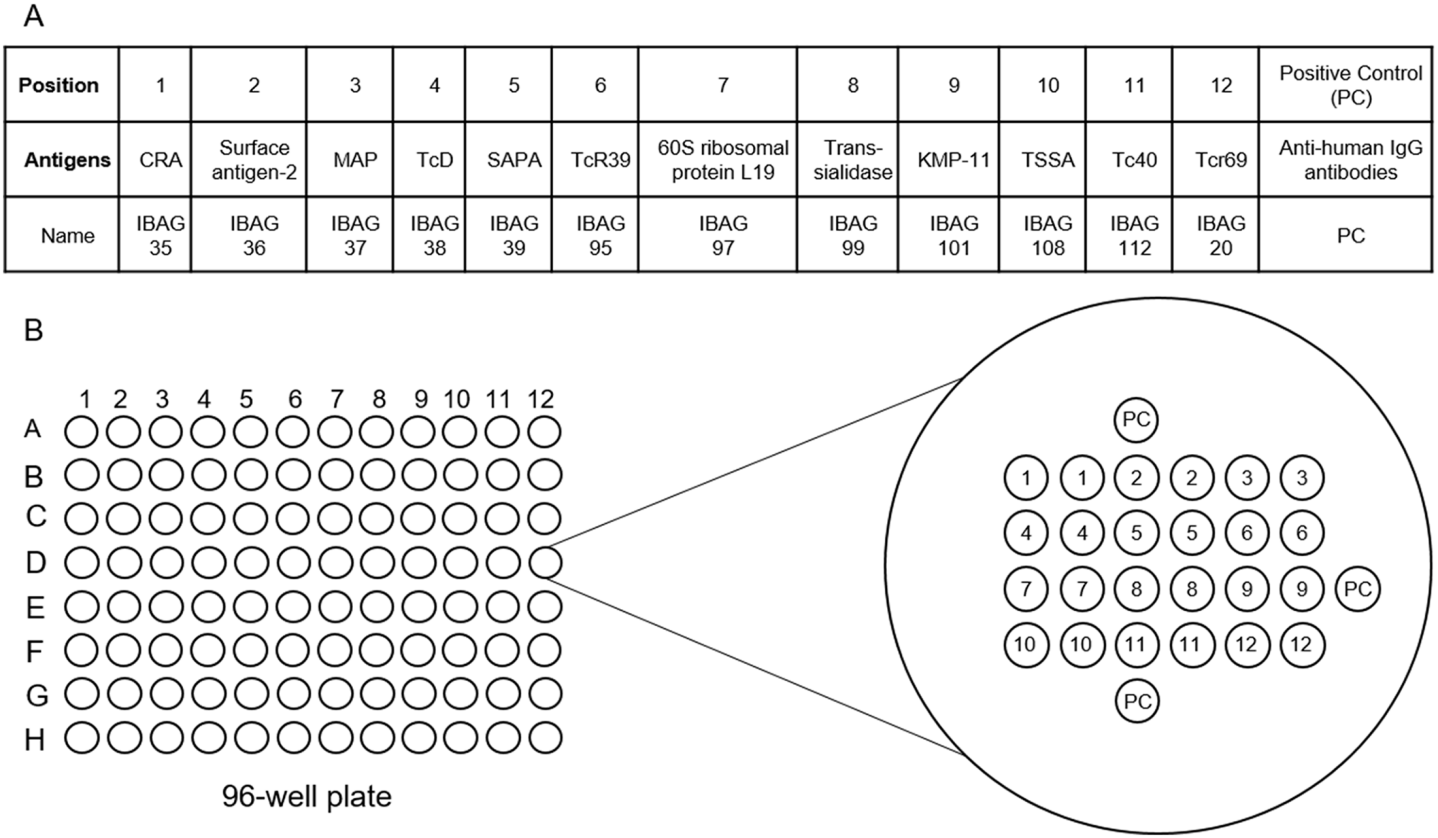
Multi-cruzi antigens map. (A) Antigens used in the assay and their respective position in the array are given together with corresponding internal reference or antigen classical names. (B) Schematic representation of the twelve antigens spotted in duplicate on the 96-well plates.

The twelve *T*. *cruzi* antigens, selected for their individual performance, were printed in duplicates in each well of a 96-well plate (Maxisorp Nunc) using a sciFLEXARRAYER printing system (SCIENION, Germany). Three spots of positive controls (PC) designed to check for the presence of human samples and enzyme conjugates were printed on the array using a precise orientation pattern (see Fig 1).

For each antigen we used a panel of negative and positive samples to optimize the concentration and obtain the best balance between sensitivity and specificity. Each antigen was diluted with carbonate buffer (pH 9.6) with concentration ranging from 70 to 130 μg/mL. Printing was performed under monitored temperature and controlled humidity conditions. Following spotting, plates were incubated overnight in the same conditions, then dried at 37°C for 2h and washed with PBST (phosphate-buffered saline with 0.05%Tween20). A blocking solution (BSA in PBS) was added to the microplate for 2h at room temperature to block nonspecific binding. Following saturation, microplates were washed with PBST. For long term storage, blocked arrayed microplates were stored at 4°C in sealed plastic bag with desiccant.

### Multi-cruzi Chagas ELISA Assay

The microplates were incubated with the blood donors or patients sera diluted at 1:50 for 1h at room temperature and washed three times with PBST. The dilution parameter was optimized during the assay development. Next, horseradish peroxidase (HRP)-conjugated goat anti-human IgG antibodies (Abliance, France) adequately diluted was added to the microplate for 1h at room temperature. The microplates were then washed three times before adding a TMB solution (SDT Gmbh, Baesweiler, Germany) for 20 min at room temperature in the dark. Then, TMB solution was removed and plates were dried at 37°C for 10 min. Each plate was imaged then analyzed using the SensoSpot Microarray Analyzers (Sensovation, Radolfzell, Germany). The software calculated the pixel intensity for each spot. In order to establish the net intensity for each antigen, we considered the mean value of the paired spots

### Statistical Analysis

Descriptive statistics are presented as frequencies for categorical variables and as mean values for continuous variables. The Mann Whitney U test was used to compare continuous variables. A cut-off value for each antigen was calculated for determining seropositivity, it represents the 95 percentile of the reactivity in pixel intensity of the blood donor population. Receiver Operating Characteristic (ROC) and statistical analyses were performed using SPSS software (SPSS version 19.0).

## Results

### Multi-cruzi Chagas Assay

Following individual validation of each antigen immunoreactivity in a classical single parameter ELISA, we selected the 12 antigens that showed the best performance properties (Fig 1A). Antigens were spotted in duplicate and organized in a 6 X 6 array in each wells of a 96-well plate (Fig 1B). Subsequent to individual calibration, antigens were spotted at various concentrations ranging from 70 μg/mL to 130 μg/mL. In addition to these antigens, we used internal positive control in triplicate (PC) to check for the reactivity in each well; the control allows confirming the serum was properly added to the well and all reagents (conjugated secondary antibody and substrate solution) were present and functional. The three positive control spots allow also space-orientation of the array in the reader.

**Fig 2:**
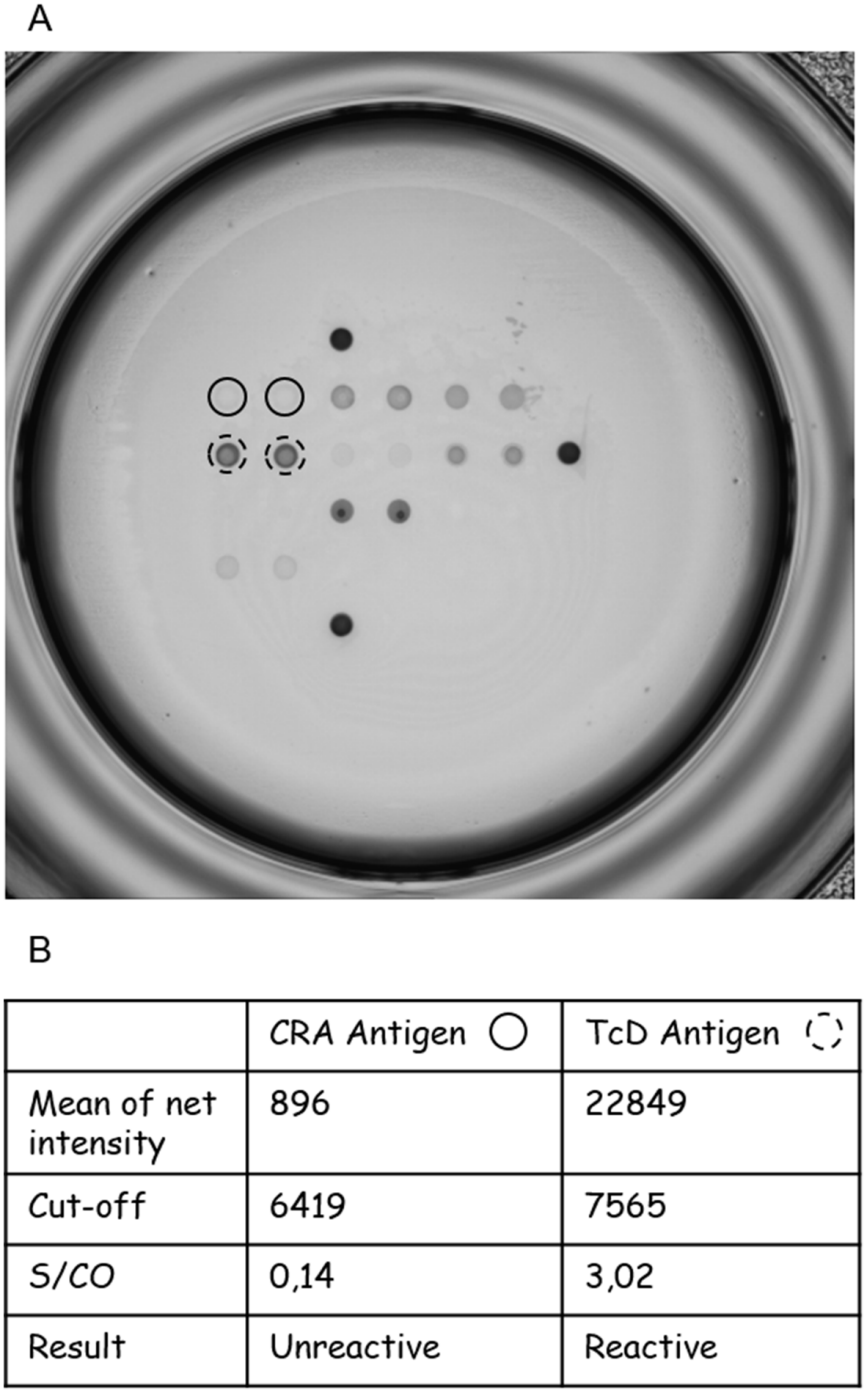
Examples of antigens reactivities on Multi-cruzi platform. (A) Scanned image showing reactivity of a positive sample (IB3491) on different antigens. CRA and TcD antigens are surrounded by a solid circle and a dotted circle respectively. (B) Values for the net intensity, cut-off and S/CO for CRA and TcD.

For each antigen, we established the net intensity (mean value of duplicated spots intensity) on the 401 samples included in the study. Data obtained on negative samples were used to establish individual cut-off values for each antigen (Table 3). Net intensity of each antigen was then converted into positive or negative reactivity according to cut-off value (Table 3). For example, the net intensity of two antigens are shown in Fig 2. The net intensity of CRA antigen (solid circles) equals 896, which is below the cut-off value for this particular antigen; therefore the sample is considered as non-reactive on CRA antigen. By contrast, for the TcD antigen, the net intensity value equals 22849, which is significantly higher than the corresponding cut-off; consequently this sample is considered as reactive on TcD antigen.

**Table 3:** Cut-off Values (net intensity for each antigen).

IBAG 35	IBAG 36	IBAG 37	IBAG 38	IBAG 39	IBAG 95	IBAG 97	IBAG 99	IBAG 101	IBAG 108	IBAG 112	IBAG 20
6419	1398	5712	7565	4745	2104	1406	709	4808	1553	957	1280

### Multi-cruzi Decision Tree Optimization

A total of 391 samples well characterized (excluding the doubtful and false-positive samples) were analyzed by Multi-cruzi and included in the analysis. All of them (negative or positive samples) were previously tested on Architect apart from the 94 “negative” samples (unscreened) originating from non-endemic regions.

**Fig 3:**
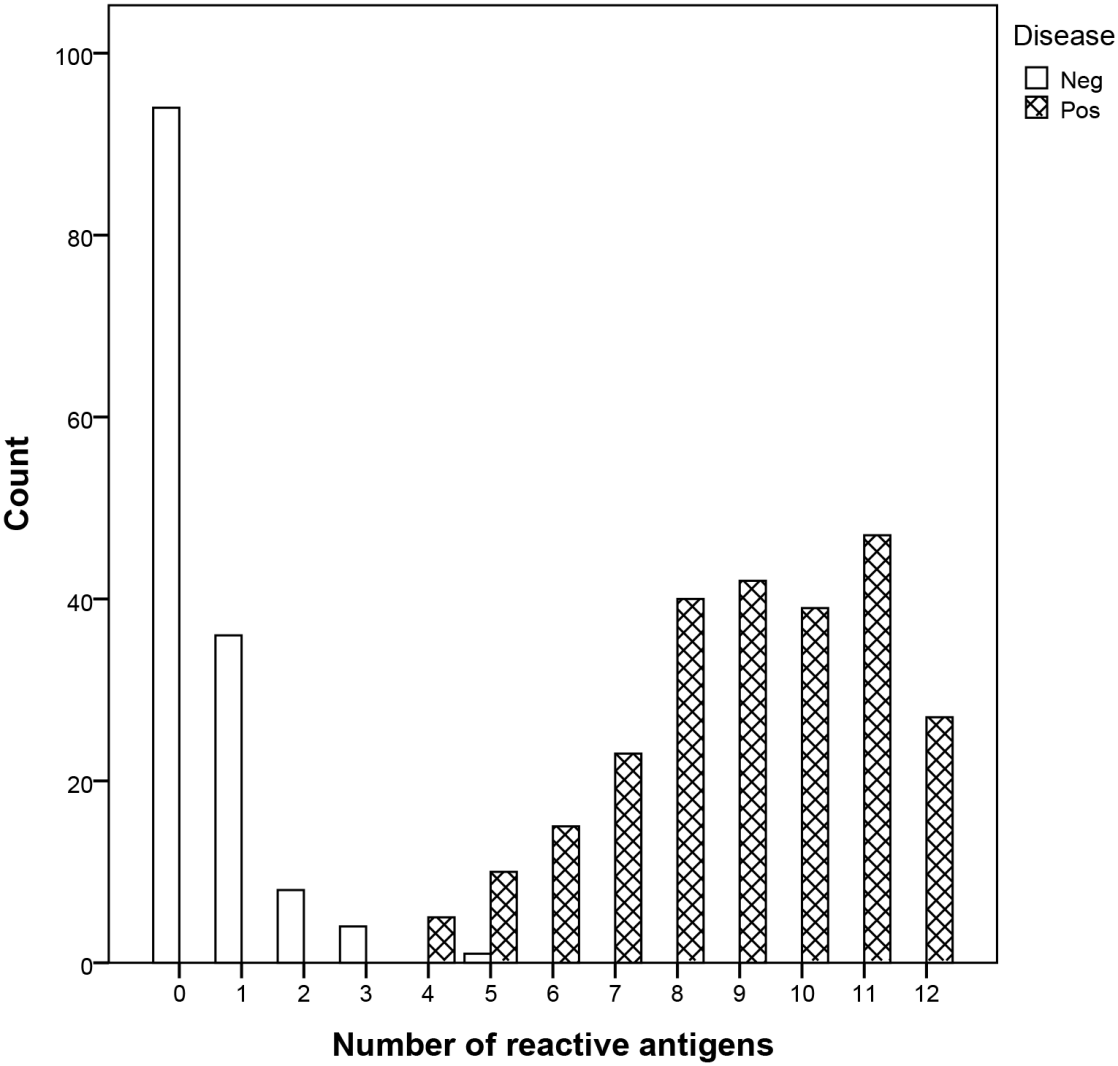
Frequency distribution of reactive antigens in non-infected or infected individuals. Number of reactive antigens out of twelve for the negative (white bars) or positive samples (shaded bars) is given as a frequency distribution. Negative samples were reactive on 0 to 5 antigens while positive samples had reactivity on 4 to 12 antigens.

**Fig 4:**
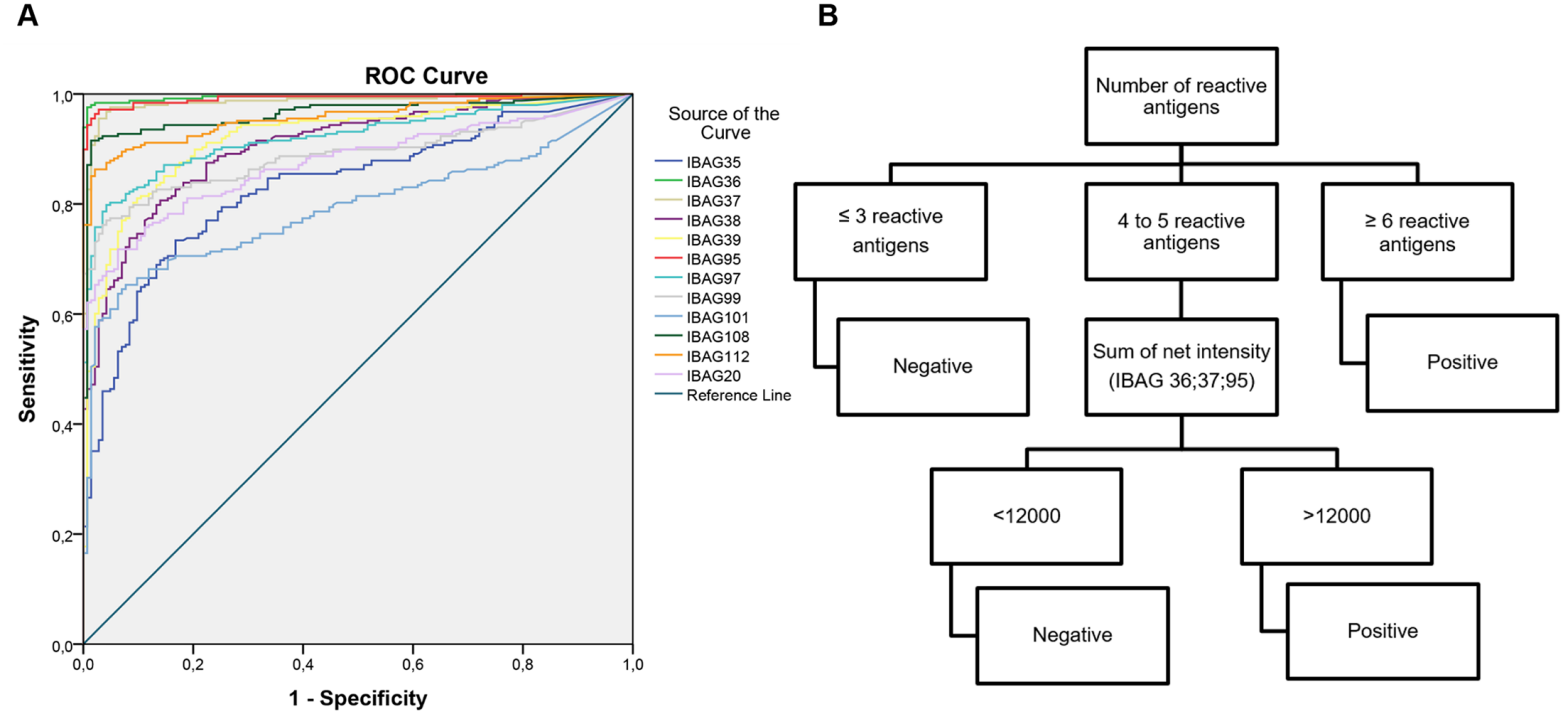
Receiver Operating Characteristic (ROC) curve and Decision tree. (A) The graph is showing the ROC curve analysis of the sensitivity and specificity of each of the 12 antigens. IBAG36-IBAG37 and IBAG95 gave the bests results. (B) The decision tree established after the first data analysis is based on the number of reactive antigens. A sample is considered as negative if reactive on up to 3 antigens and positive if reactive on at least 6 antigens. When reactive on 4 or 5 antigens the sample is further analyzed: the Sum of intensities on three antigens (IBAG36-37-95) is measured and the 12 000 raw value is used as a threshold.

In first instance, we analyzed 248 confirmed positive samples, 94 unscreened and 49 screened negative samples. Initially, intensity data were analyzed using logistic regression and multivariate analysis to propose the most optimal classification of the samples. Ultimately, this analysis guided us towards a more simplified and intuitive decision tree that we used in this study. For each population we observed the frequency of positive and negative samples in relation to the number of reactive antigens (see Fig 3, for each serum we determined the reactivity on each antigen and established the number of reactive antigens). Samples were distributed in three categories: a category with reactivity on 0 to 3 different antigens (n = 142, (99.3%) considered as negative), a category with reactivity on at least 6 different antigens (n = 233 (94%, positive samples)). The third category considered as indeterminate included sixteen sera with that reacted on 4 or 5 different antigens. Using Receiver Operating Characteristic (ROC) analyses we identified three antigens that had area under the curve values above 0.980 (Surface Antigen-2, TcR39 and MAP; Fig 4A). When we measured the Sum of intensities on these three antigens, we observed a threshold of 12000 that can clearly split this category into negative (n = 1 with a Sum of intensities <12000) and positive samples (n = 15 with a Sum of intensities >12000; Fig 4B).

Finally, the Multi-cruzi assay was able to correctly classify 248 of 248 positive samples and 143 out of 143 negative samples using the proposed decision-tree algorithm.

### Correlation between PCR Results and the Multi-cruzi Test

**Fig 5:**
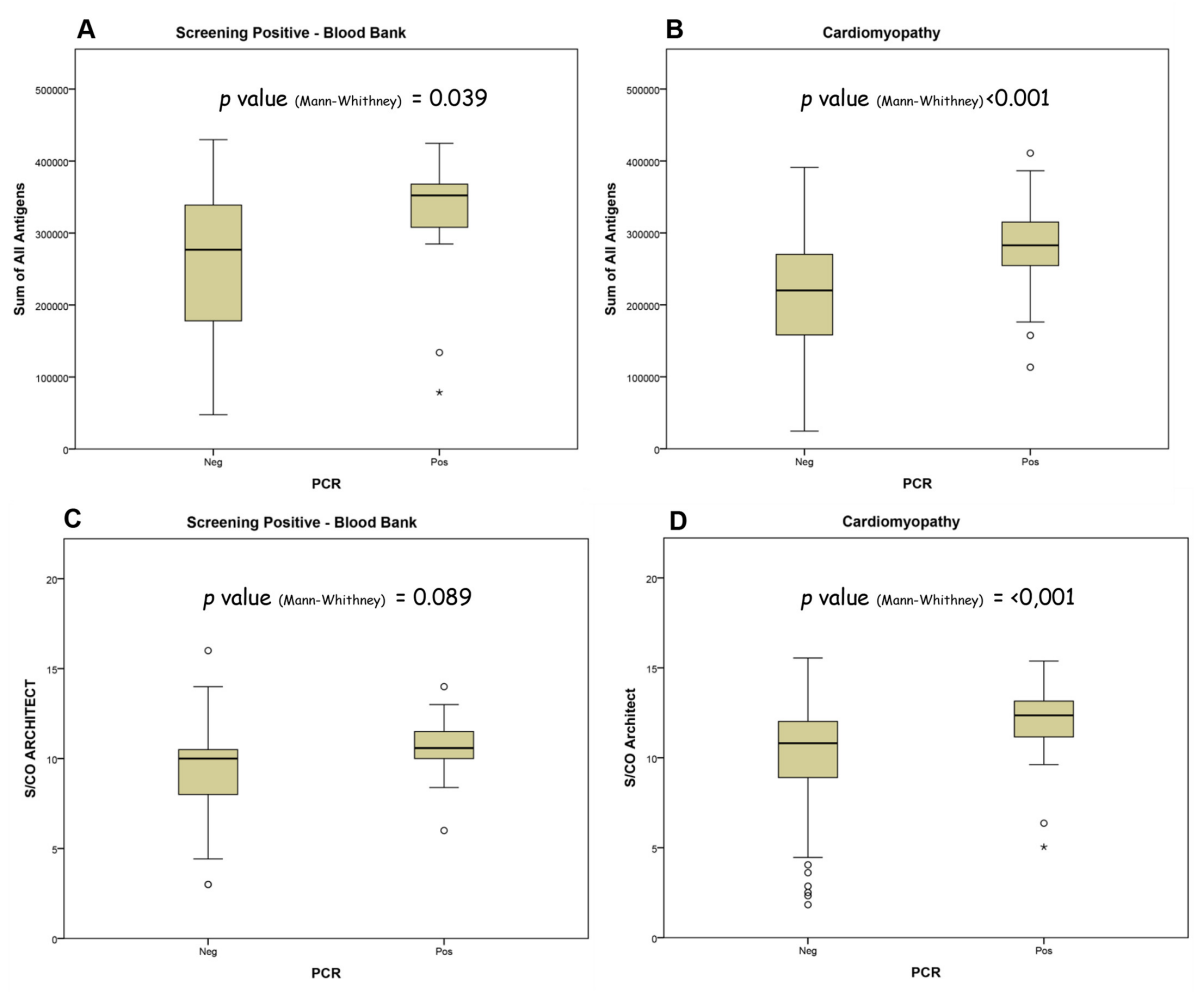
Correlation between PCR results and Multi-cruzi or Architect ELISA on samples from Blood donors or Cardiomyopathy cohort. (A and B) For infected blood donors (A) or Cardiomyopathy cohort (B), mean raw values of the sum of all antigens obtained on Multi-cruzi assay are given relative to *T*. *cruzi* PCR status. Individuals from the PCR positive group gave significantly higher values on the sum of all antigens in both cohorts. (C and D). The graphs S/CO values obtained on Architect ELISA in samples from blood donors (C) or Cardiomyopathy cohort (D) are given relative to *T*. *cruzi* PCR status. Individuals from the PCR positive group gave significantly higher values on the Architect ELISA for Cardiomyopathy patients but for positively screened blood donors. In all figure, mean and extreme outliers are represented by a circle or a star respectively.

Among the 200 cardiomyopathy samples, 194 were characterized as Negative (n = 129; 67%) or Positive (n = 65; 33%) by PCR. Additionally, among the 48 screening-positive blood donors, 34 were PCR negative (71%) and 14 were PCR positive (29%; Table 1). PCR techniques were different for both populations (see Material and Methods). For both groups, the mean Sum of all antigens’ intensities were significantly higher among PCR positive as compared to PCR negative samples (p = 0.039”Screening Pos” Fig 5A and p<0.001 “Cardiomyopathy”; Fig 5B). By contrast, we did not observe a significant difference of mean S/CO obtained with the ARCHITECT (Abbott) among PCR positive and PCR negative samples for “Screening Pos” group (p = 0.089 “Screening Pos” Fig 5C) but we observed a significant difference for “Cardiomyopathy group” (p<0.001 “Cardiomyopathy” Fig 5D).

### Resolving Screening Discrepant Samples with Multi-cruzi Assay (n = 10)

In order to assess the performance of Multi-cruzi assay, we tested three doubtful samples and seven false-positive samples.

**Fig 6:**
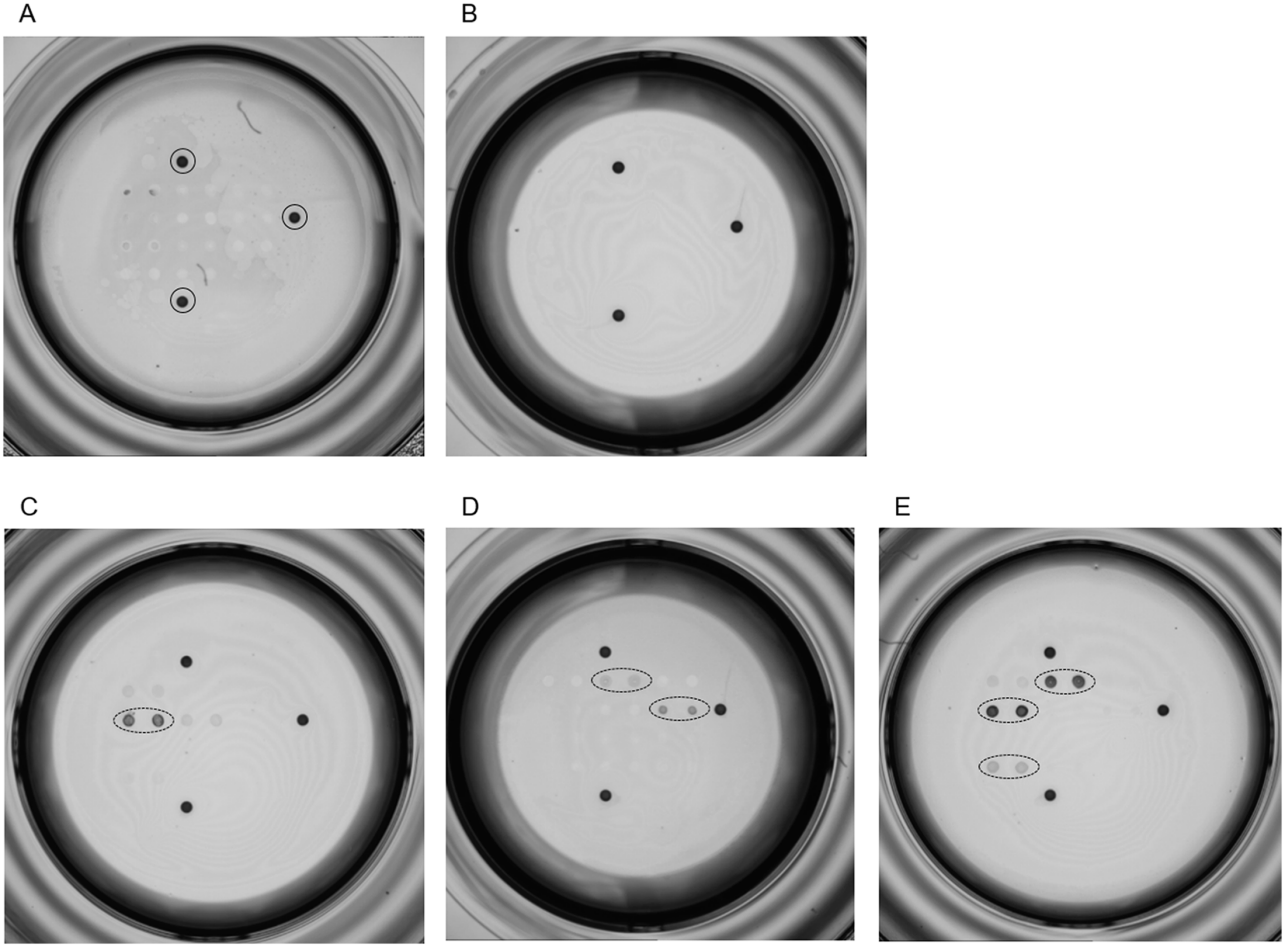
Pictures of false-positive samples. Scanned images show the results obtained on Multi-cruzi assay with false-positive samples. (A and B) IB3522 and IB3520 show no specific reactivity. In (A) background staining is typical of a sticky sample. Positive controls are surrounded by a solid circle (A). (C to E) Positive spots according to S/CO are surrounded by a dotted circle. IB3523, IB3521 and IB3524 samples are reactive on one, two or three antigens (C, D and E respectively).

False-positive samples turned out to be non-reactive or reactive on maximally three antigens (Fig 6 shows few examples). According to the proposed decision tree, all presumed false-positives were classified as likely negative (Table 2). For IB3522, the spotted antigens remained uncolored (absence of specific antibodies) while a dark background spreads all over the plastic surface of the microwell (nonspecific binding). IB3522 is thus a classical example of “sticky sample” giving false reactivity on standard ELISA technics.

About doubtful samples, IB3515 and IB3516 were interpreted as presumably negative according to the decision algorithm (Table 2). However, IB3517 was regarded as likely positive with four reactive antigens and a sum of IBAG 36-37-95 superior to 12000.

Finally, the false-positive and two doubtful samples were all considered as presumably negative on the Multi-cruzi because they were reactive on less than four antigens. However, one doubtful was considered putatively positive. These different examples illustrate the potential of the Multi-cruzi test to solve ELISA’s classical problems and its ability to further classify doubtful samples.

## Discussion

In this study, we evaluated a new multiplex assay for the detection of different specific antibodies against *T*. *cruzi* in sera of patients with Chagas disease. Diagnostic and screening tests are essential tools that help determining if a patient or a blood donor has an infection or not. In the context of an infection, like for Chagas disease, serological assays can even be more essential as many parasite carriers may not present any symptoms. For example, a healthy pregnant women originating from Latin America, should be tested for *T*. *cruzi* infection in order to anticipate parasite transmission to her infant and eventual implementation of the adequate treatment to the neonate. For several infectious diseases, diagnostic information is based on serological reactivity as PCR-based tests are only sensitive in the presence of the causative pathogen. In case of positive results on a screening test, a confirmatory test is typically performed to avoid incorrect decision due to falsely reactive samples. So far, confirmation of Chagas Disease has not been standardized. Shah et al described a dot Blot assay, including the four Fusion Protein used in the ARCHITECT automat, but its usage does not seem to be generalized so far [12]. Furthermore, as the same antigens are present in the ARCHITECT and the ESA blot, their sequential utilization may introduce a bias by giving redundant information and does not comply with the requirements of a complete diagnostic

Oelemann et al. [16] reported the development of serological confirmation assay for Chagas disease: the INNO-LIA Chagas that used the multiparametric approach to resolve indeterminate results. In the present work, we have extended the multiparametric approach to further explore the diversity of humoral immune response and attempt to classify the samples beyond positive and negative and possibly into different clinical groups. The National Consensus on Chagas disease (Brazil) suggested the use of another assay: the TESAcruzi [20]. TESAcruzi is a Western blot utilizing *T*. *cruzi* tripomastigote secreted and excreted antigens. However, Caballero et al. showed that the use of recombinant antigens or synthetic peptides are more specific than those using crude extracts from *T*. *cruzi* epimastigote forms [21]. Following these observations we used well characterized synthetic antigens for which the linear amino-acids sequences were optimized in terms of size and composition to avoid cross-reactivities.

After selecting a panel of twelve antigens, we optimized the decision tree to separate the patients from uninfected blood donors’ samples. The interpretation algorithm is optimized to obtain a 100 percent concordance with the well-characterized seropositive and seronegative samples used in this study. Multi-cruzi technology by individually revealing reactivity on the antigens would allow solving some of ELISA’s problems such as “sticky” or false-reactive samples. Another concern for *T*. *cruzi* confirmation is cross-reactivity with *Leishmania sp*. In the proposed Multiplex test, we use a large number of antigens that were pre-selected for their sensitivity and specificity attributes on *Trypanosoma cruzi* infected panels. The probability of facing multi-reactive samples that are not *Trypanosoma cruzi* infected is reversely correlated to the number of reactive antigens. In other words, a *Leishmania* sample is likely to react on one, two or three antigens, but becomes unlikely to react on four or more *T*. *cruzi* antigens. Moreover, in the cases of doubtful samples, meaning reactive on two ELISAs, Multi-cruzi assay allowed defining their status: out of three samples, two were likely negative and one was probably positive. These donors were all originating from the endemic zone (Bolivia or Argentina). For the doubtful samples that were found negative, we suggest two hypotheses: these donors were not infected by *T*. *cruzi* or infected but spontaneously cured [22] [23]. The doubtful sample that were found positive are possibly infected by *T*. *cruzi* yet to be confirmed. A longitudinal follow-up of these samples, would allow acceptation or rejection these assumptions. Interestingly, the two infected population used in this study are different: one was made of samples from patients suffering cardiomyopathy and the second made of blood donors that screened positive. Moreover geographical origins of the samples was also diverse as one cohort came from Brazil and the blood donors have various origins (mainly Bolivia). These observations reinforce the robustness of our test that could be used in the future either in the context of clinic or the blood screening, and on patients of various geographical origins.

In the absence of an appropriate confirmation assay for Chagas disease, confirmation today is mainly derived from repeated reactivity on a variety of distinct screening tests, and when results are still inconclusive, PCR is also used to circumvent a potential transmission of circulating parasite. PCR is extremely specific (100%) but unfortunately its sensitivity is not good enough to be used as a diagnostic tool for *T*. *cruzi* detection (between 50 to 90% according to Brasil [9]) as the level of circulating parasites in blood decreases during the chronic phase. Interestingly Sabino et al [19] observed that the antibodies levels (Ortho ELISA) in patients are correlated to qPCR results. We also observed that antibodies levels (Sum of all antigens) was higher for PCR positive than for PCR negative samples in all studied groups with Multi-cruzi. However, with ARCHITECT ELISA, the correlation between PCR results and antibodies levels was only observed in the “Cardiomyopathy” group.

Altogether, our findings may suggest that the “sum-of–all-antigens” results obtained with the Multi-cruzi could correlate with parasitemia level in certain patients (See Fig 5) and may thus indicate the status of parasite clearance in a longitudinal follow-up.

Multiplex assays offer several advantages. First of all, using a small quantity of sample, a large quantity of results are obtained. Indeed, with our multiplex assay we were able to obtain results on twelve different antigens using few microliters of sample and in few hours [24]. Moreover the possibility to print a major amount of antigens, (an array of 12 x 12 spots) can permit to establish a complete immunological signature for each patients. Secondly, the Multi-cruzi assay has an internal control embedded for each individual sample. This is a security that cannot be obtained with classical ELISA procedures. Finally, Sánchez Negrette et al. [25] have shown that two antigens classically integrated in diagnostic assays, revealed faster regression trends in response to treatment in comparison with the global ELISA signals including those antigens. The multiplex approach by giving a discrete analysis of each antigen rather than a single composite response is an extremely sensitive methodology that could be promising to anticipate disease evolution (Cardiomyopathy or Intestinal) or monitor Chagas disease therapy.

The results described in this study indicate that the Multi-cruzi is a reliable confirmatory assay for the serodiagnosis of Chagas disease, with the potential to discriminate false-positive results and sticky samples. This new multiplex approach will be useful to establish the serological response pattern in each patient. To clinically validate the performance of the Multi-cruzi assay, additional studies, including typical falsely reactive samples such as Leishmaniosis and a larger number of routine testing for Chagas disease samples, should be conducted. Upon further testing, the proposed decision tree may still be subject to amendments and fine-tuning.
